# Depletion of adult neurogenesis using the chemotherapy drug temozolomide in mice induces behavioural and biological changes relevant to depression

**DOI:** 10.1038/tp.2017.68

**Published:** 2017-04-25

**Authors:** M Egeland, C Guinaudie, A Du Preez, K Musaelyan, P A Zunszain, C Fernandes, C M Pariante, S Thuret

**Affiliations:** 1 Department of Psychological Medicine, Maurice Wohl Clinical Neuroscience Institute, Institute of Psychiatry, Psychology and Neuroscience, King's College London, London, UK; 2 Department of Basic and Clinical Neuroscience, Maurice Wohl Clinical Neuroscience Institute, Institute of Psychiatry, Psychology and Neuroscience, King's College London, London, UK; 3 MRC Social, Genetic and Developmental Psychiatry Centre, Institute of Psychiatry, Psychology and Neuroscience, King's College London, London, UK

## Abstract

Numerous studies have examined links between postnatal neurogenesis and depression using a range of experimental methods to deplete neurogenesis. The antimitotic drug temozolomide (TMZ) has previously been used successfully as an experimental tool in animals to deplete adult neurogenesis and is used regularly on human patients as a standard chemotherapy for brain cancer. In this study, we wanted to evaluate whether TMZ as a model for chemotherapy treatment could affect parameters related to depression in an animal model. Prevalence rates of depression in patients is thought to be highly underdiagnosed, with some studies reporting rates as high as 90%. Results from this study in mice, treated with a regimen of TMZ similar to humans, exhibited behavioural and biochemical changes that have relevance to the development of depression. In particular, behavioural results demonstrated robust deficits in processing novelty and a significant increase in the corticosterone response. Quantification of neurogenesis using a novel sectioning method, which clearly evaluates dorsal and ventral neurogenesis separately, showed a significant correlation between the level of ventral neurogenesis and the corticosterone response. Depression is a complex disorder with discoveries regarding its neurobiology and how it relates to behaviour being only in their infancy. The findings presented in this study demonstrate that chemotherapy-induced decreases in neurogenesis results in previously unreported behavioural and biochemical consequences. These results, we argue, are indicative of a biological mechanism, which may contribute to the development of depression in patients being treated with chemotherapy and is separate from the mental distress resulting from a cancer diagnosis.

## Introduction

During the past decade, researchers in psychiatry, neuroscience, as well as the pharmaceutical industry have placed intense interest on the connections between depression, adult neurogenesis and antidepressants. A major hurdle, however, in elucidating these connections is a lack of translational studies which confirm in humans, that which has already been done in *in vitro* and animal studies. Recently, it has been established that the level of hippocampal adult neurogenesis in humans is considerable throughout life and may be even greater than that of rodents, making the relevance of adult neurogenesis in human diseases an even more pressing question.^1, 2^ Obvious ethical implications of experimentally decreasing adult neurogenesis in humans makes human studies on the function of neurogenesis impossible. However, the antimitotic mechanisms of many of the life-saving chemotherapy treatments for cancer have the likely side effect that they decrease adult neurogenesis.^3^ Studying the biological and behavioural effects of chemotherapy in animal models may be a way to begin a translational approach into understanding how chemotherapy-induced decreases in adult neurogenesis may affect humans and specifically, how it may affect the development of depression.

A recent meta-analysis of the prevalence of depression in cancer patients show that brain cancer is the subtype, which is mostly associated with depression.^4^ Studies show that the prevalence rates in this type of cancer are 28%. However, depression in cancer patients is thought to be highly underdiagnosed with one study pointing out that 93% of patients self-reported symptoms of depression, whereas only 22% were actually classified as depressed at some point during treatment.^5^ The devastating nature of cancer makes it difficult to discern any effects of psychosocial stress from potential side effects of the treatment and was not examined in any of the mentioned studies. However, harnessing the translational relevance using animal models may help isolate any biological effects of these drugs relevant to depression. Temozolomide (TMZ) is an antimitotic drug that, due to its low levels of non-central nervous system toxicity and increased survival rate, has become the standard form of chemotherapy for brain cancers.^6^ TMZ has also recently been developed as an experimental tool to decrease adult neurogenesis in animals using a similar cyclic protocol used in the clinic, which was found in rodents to reduce levels of adult neurogenesis by 80% at the end of treatment.^7^ Specifically, several studies have used TMZ to examine different functions in animal models thought to be neurogenesis dependent including aspects of spatial learning and addiction.^7, 8, 9, 10, 11^ These studies have not, however, addressed any potential links between neurogenesis depletion as a result of TMZ with depression. Owing to its low toxicity in animals and published anti-neurogenic effects, TMZ is a good drug to model the effects of chemotherapy.

The role that adult neurogenesis has in the development of depression is continuing to be defined.^12, 13^ The results from numerous studies in which neurogenesis is depleted using various methods show the development of some depressive-like symptoms in some studies but not others,^14, 15^ indicating that the idea that a decrease in neurogenesis simply results in immediate depression is unlikely. However, a consistent finding is that new neurons are required for antidepressant efficacy.^16, 17, 18^ The neurobiological mechanisms resulting in the numerous symptoms of depression are still largely undefined as is the potential role of adult neurogenesis in these mechanisms. A recent review in the field though suggests that the connection between neuropsychiatric disorders and dysregulated hippocampal neurogenesis is beyond correlation or epiphenomenon.^12^ An often-repeated caution to the field of depression research is the lack of suitable methods with which to evaluate depression in experimental models, indicating that results using established paradigms should be interpreted carefully.

Definitions of anatomy may be one important factor that has previously been underestimated in many studies and may contribute to these conflicting results.^19^ It is becoming increasingly evident that there are differences, including anatomical, functional and molecular distinctions, along the dorsoventral (septo-temporal) axis of the hippocampus.^20^ Furthermore, adult-born neurons in the ventral dentate gyrus have previously been shown to mature at a slower rate than those of the dorsal.^21^

In this study, we have combined knowledge about the dynamics of adult neurogenesis with a new sectioning method that allows greater dorsoventral segregation in addition to the newly developed pharmacological method using TMZ to deplete adult neurogenesis in mice. With these tools, we aimed to investigate whether the high rates of depression seen in cancer patients undergoing chemotherapy can partly be attributed to biological changes as a result of a decrease in adult neurogenesis.

This study has particular relevance to cancer patients being treated with TMZ but as all forms of chemotherapy have similar cytostatic effects, there is a pertinent relevance of this study to all forms of chemotherapy. By examining the behavioural and biological changes of chemotherapy in animals, this study may give some of the most translational idea to date of what can potentially be occurring in humans with a chemotherapy-induced depletion of adult neurogenesis.

## Materials and methods

### Animals

Eight-week-old male C57BL/6J mice were purchased from Charles River Laboratories (Kent, UK). The mice were group housed (four per cage) in standard cages measuring 32 × 16 × 14 cm. The housing rooms were kept at an ambient temperature (21±2 °C) and light (light/dark cycle with white lights on from 0800 to 2000 h), with food (Rat and Mouse No. 1 Diet; Special Diet Services, Essex, UK) and tap water available *ad libitum*. To avoid the disruptive effect of cage cleaning on behaviour, Sawdust (Litaspen premium), nesting materials (Sizzlenest; Datsand, Manchester, UK) and a cardboard shelter (LBS Biotech, Horley, UK) were changed once every 2 weeks, but never on the day before or the day of testing. All housing and experimental procedures were performed in compliance with the U.K. Home Office Animals Scientific Procedures Act 1986. The work was carried out under license (PPL: 70/7184) and all efforts were made to minimize animal suffering and to reduce the number of animals used.

### TMZ treatment

**Figure 1:**
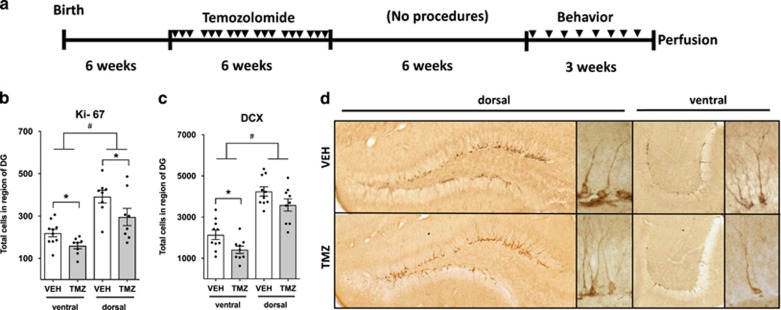
TMZ treatment induces sustained decreases in adult neurogenesis. (**a**) The experimental design was performed as visually represented. The mice were treated (25 mg kg^−1^ temozolomide (TMZ)) in cycles of 3 days TMZ or vehicle (VEH) and 4 days recovery (*n*=10). (**b**) Significant decreases in cell proliferation using ki-67 were found in both dorsal and ventral dentate gyrus (DG) of TMZ-treated mice (*P<0.05, one-way analysis of variance (ANOVA)). Significantly less cell proliferation (ki-67)/volume is found in the ventral vs dorsal DG (^#^*P*<0.001, two-way ANOVA) (**c**) A significant decrease in DCX-expressing immature neurons was present in ventral (*P<0.05, one-way ANOVA) but not dorsal DG. Significantly less differentiation (DCX)/volume is found in the ventral vs dorsal DG (^#^*P*<0.001, two-way ANOVA) (**d**) Micrographs of hippocampus labelled with DCX presenting representive differences between the ventral and dorsal regions in staining density. High magnification of representative DCX-positive cells demonstrating no differences in morphology of DCX+ cells between treatment groups.

The mice belonging to the TMZ treatment group (*n*=10) were injected with TMZ (T2577, Sigma, Gillingham, UK) at a concentration of 25 mg kg^−1^ (intraperitoneal injection, 2.5 mg ml^−1^ in 0.9% NaCl) while controls (*n*=10) were injected with the 0.9% NaCl vehicle. To mimic the cyclic treatment administered in the clinic, the animals were injected on 3 consecutive days every week for a total of 6 weeks (Figure 1a). The mice were then allowed to recover for a period of 6 weeks. Behavioural tests began after the recovery period and ran over 3 weeks with the exception of the novelty-suppressed feeding (NSF) test done before and after recovery.

### Behaviour

The mice were 10 weeks old at the start of testing and the tests were recorded using a camera positioned above the test arenas and the movement of each mouse tracked using EthoVision software (Noldus Information Technologies bv, Wageningen, The Netherlands; http://www.noldus.com/site/doc200403002).

### Novelty-supressed feeding

Novelty-suppressed feeding was measured according to previous protocols.^22^ The testing setup consisted of a rectangular plastic box (32 × 16 × 14 cm), the floor of which was covered with fresh bedding for each mouse. The mice were food-deprived for 24 h before behavioural testing. At the time of testing, a food pellet was placed on a square piece of white cardboard (cage label) in the centre of the box. An animal was placed in a corner of the box and the latency to eat the pellet was measured. Food consumption over a 24 h period was then measured to control for changes in appetite.

### Sucrose preference

The mice were individually housed for 2 days with *ad libitum* access to food and a choice of two bottle sippers, one containing tap water, the other containing a 1% sucrose solution. The position of the bottles alternated between day one and day two, to prevent side bias. Consumption was measured 24 h and 48 h after the start of the test. Sucrose preference was measured as sucrose solution consumption/total fluid consumption per day.

### Open field

The mice were placed facing the wall of a circular open-field arena (40 cm diameter) and allowed to freely explore the arena for 10 min according to previous protocols.^23^ A small lamp placed on the test room floor provided dispersed lighting (25 lux). In the EthoVision software, a central circle of equal distance from the periphery, defined as the ‘central zone', was virtually drawn within the arena. The frequency of entries into, and the time spent in, the central zone of the arena were extracted in EthoVision, in addition to the total distance travelled (cm).

### Elevated plus maze

The mice were tested according to previous protocols.^23^ The maze apparatus was made of acrylic and consisted of two opposing closed arms (30 × 5 cm), which were enclosed by 15 cm high grey acrylic walls, and two opposing open runways (30 × 5 cm), with 0.5 cm high grey acrylic ledges around the open arms. The maze was elevated 40 cm from the ground on a transparent acrylic stand. Light intensity in the open arms was 40 lux, compared with 20 lux in the closed arms. The mice were placed on the central platform, facing one of the open arms, and left to explore the maze undisturbed for 5 min. The number of entries into, and the time spent in, the open and closed arms were scored using EthoVision software. Zones to measure entry/exit were defined in EthoVision as an area where roughly all four paws of the average mouse had entered an arm.

### Forced swim test

The mice were placed for 6 min in a transparent cylinder measuring 15 cm in diameter and filled with room temperature water to a depth of 40 cm, as previously described.^22^ The activity in the trial was recorded using a camera positioned perpendicular to the forced swim test and behaviours were scored manually from the videotape using EthoVision; struggling (defined as a vertical posture with limb movement), swimming (defined as a horizontal posture with limb movement), paddling (defined as horizontal posture with minimal movement in one limb). Mean duration and latency of immobility were extracted from the manual scores. (faecal boli were removed at the end of each trial).

### Tail suspension test

The mice were individually suspended by the tail from a horizontal bar 35 cm from the floor, using adhesive tape (distance from tip of tail was 2 cm). Trial duration was 6 min and each trial was videotaped and subsequently scored for the number of seconds spent immobile.^22^

### Immunohistochemistry

Directly after the last day of testing, the mice were prepared for fixation by first being deeply anaesthetized with pentobarbitol (euthatal, 200 mg kg^−1^ body weight) and then perfused with cold phosphate-buffered saline (PBS; 0.1 m; pH 7.4) followed by 4% paraformaldehyde. The brains were removed, stored in fixative overnight, transferred into 30% sucrose and sectioned as described in the 'Results' section. The sections obtained using this method were stored in PBS/sodium azide at 4 °C until staining was performed. The primary and secondary antibodies were diluted in PBS saline containing 0.1% Triton X-100 and 3% normal goat serum (NGS; Vector Laboratories, Peterborough, UK, S-1000). Immunohistochemistry stainings to visualize stages of adult neurogenesis were performed using 3,3-diaminobenzidine. Briefly, the sections were permeabilized (15 min. in 1% Triton X-100/0.1 m PBS), blocked against endogenous peroxidases (20 min with 3% H_2_O_2_/0.1 m PBS) and then incubated with NGS for 1 h followed by the primary antibody. For primary antibody staining, the sections were incubated overnight in either anti-Ki-67 (Abcam, Cambridge, UK, AB15580; 1:1000 in 10% NGS) or anti-DCX primary antibody (Abcam, AB18723; 1:1000 in 10% NGS). The sections were then incubated in anti-rabbit secondary biotinylated antibody (Vector Laboratories, BA-9400; 1:250 in 3% NGS 2 h at 20 °C). A signal amplification step was then performed by incubation in ABC reagent (Vector Laboratories, PK-6100, Vectastain Elite ABC-Peroxidase Kits; 1:1000; for 1 h) followed by a reaction with 0.05% 3,3-diaminobenzidine tetrahydrochloride/0.01% hydrogen peroxide (Sigma, D5637-5G) in PBS. Finally, the sections were dehydrated and mounted on polylysine-coated slides (Histolab), dried and coverslipped using DPX (Sigma, 44581).

### Stereology

A stereological method was used to quantify the total number of immunopositive cells using An Axioskop 2 MOT Zeiss microscope and a semiautomatic stereology system (StereoInvestigator, Microbrightfield, Williston, VT, USA). With this system, the region of interest was manually traced and the total number of immunopositive cells in the region of interest counted. This was done for every sixth section throughout the section of hippocampus (dorsal or ventral). Dorsal sectioning resulted in an average of 8 sections per brain while the dorsal sectioning resulted in an average of 6 (total 14 sections per brain). The software then estimated the total number of positive cells per section of dentate gyrus. Because Ki-67-positive cell are found in low numbers at this age, sampling with the StereoInvestigator system was not possible with a reasonable coefficient of variance.^24^ Similar to previously published protocols,^24, 25^ all individual Ki-67-positive cells were manually counted using × 20 objective (Leitz, Leica Microsystems, Milton Keynes, UK) on an Axioskop 2 MOT Zeiss microscope in every sixth hippocampal section (dorsal or ventral) and this number used to estimate the total number of Ki-67-positive cells per section of dentate gyrus.

### Corticosterone assay

Approximately 50 μl of whole blood was collected via tail sampling exactly 24 h before forced swim test (baseline or pre-stress measure) between 1000 and 1200 h and again 30 min after the forced swim trial (post-stress measure). Forced swim testing was performed between 1000 and 1200 h. Blood collection was completed within 120 s after removing each mouse from its cage by a small incision using a scalpel at the base of the tail. Blood obtained was collected into potassium-EDTA microvette CB 300 tubes (Sarstedt, Nümbrecht, Germany), stored on ice and centrifuged with 12 000 r.p.m. at 4 °C for 10 min to separate out the plasma. Blood plasma was stored at –20 °C. Plasma corticosterone levels were determined in duplicate from 20 μl of plasma using commercially available enzyme immunoassay kits (Enzo Life Sciences, Lausen, Switzerland); sensitivity 30 pg ml^−1^.

### Statistical analysis

The mice with different treatments (TMZ or vehicle) were compared primarily using two-tailed unpaired Student's *t*-test when it was appropriate. When additional variables were present, one-way analysis of variance followed by Newman–Keuls *post hoc* test was used. Statistical significance was defined as *P*<0.05. Statistical analyses of the data were carried out with GraphPad Prism 6.

## Results

### Novel sectioning method for greater segregation of the dorsoventral dentate gyrus

**Figure 2:**
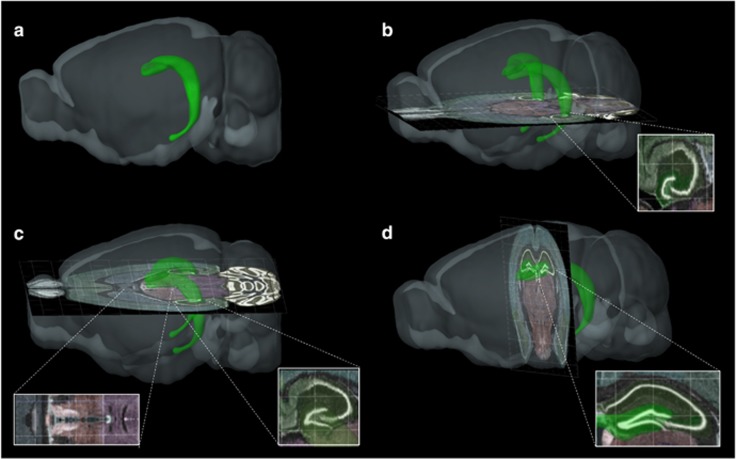
Novel sectioning method. (**a**) The J-shaped rodent hippocampus (green) makes it impossible to get uniform sections from both the dorsal and dentate gyrus (DG) when sectioning in traditional coronal fashion. (**b**)To obtain sections for the ventral hippocampus, the brain was frozen to the stand on it dorsal side and then sectioned at 40 μm along the axial plane from the ventral side of the brain toward dorsal producing uniform ventral DG sections (inset, **b**). (**c**) When reaching the beginning of the third ventricle (left inset, **c**) marking the mid-section of the DG, the specimen is detached, reoriented and attached by its caudal side in order to section the dorsal hippocampus along the coronal plane (inset, **d**). For a one-in-six quantification, this method gave approximately eight similar volume sections from both the dorsal and ventral regions. These sections were then analysed using stereology.

The current gold standard for quantification of adult neurogenesis is via stereological quantification of coronal sections. Owing to the ‘J'-like shape of the mouse hippocampus (Figure 2a), it is not possible when sectioning coronally to get numerous, similar and thus representative sections of the ventral dentate gyrus.^19^ To achieve a more accurate measure of potential differences in ventral vs dorsal quantifications, a new sectioning method was therefore devised and used in this study (see Figures 2a–d for the description of method).

### TMZ induces a sustained decrease in adult neurogenesis in the ventral dentate gyrus

Previous studies^7, 8^ have examined processes mainly associated with the dorsal hippocampus and accordingly used a 4-week TMZ treatment protocol to deplete an entire cohort of new neurons as maturation of dorsal hippocampal neurons is roughly 4 weeks. However, the maturation rate of the mood-associated ventral dentate gyrus is slightly slower and is approximately 6 weeks.^21^ A treatment paradigm was therefore devised here in which adult male C57Bl6 mice were treated for a period of 6 weeks with 25 mg kg^−1^ TMZ followed by a 6-week period in which the mice recovered from any adverse effects of the drug (Figure 2a). Our previous pilot studies demonstrated that at 6 weeks after cessation of TMZ treatment, the mice have still a 60% decrease in the levels of DCX+ immature newborn neurons (data not shown).

The levels of neurogenesis were examined 9 weeks after cessation of the TMZ treatment (Figure 1a). The comparison of the results from quantification of control mice alone demonstrated that the volume controlled levels of both cell proliferation (Ki-67) and differentiation (DCX) were significantly less in the ventral vs dorsal dentate gyrus (Figure 1b and c). The cell proliferation was found to be 49% less in the ventral dentate gyrus whereas differentiation was 51% less. Statistical analysis of this difference is highly significant for both markers using two-way analysis of variance with region as a factor (*P*<0.001). This clearly indicates that in brains from this study there are comparatively fewer neurogenic total cells in the ventral dentate gyrus than in the dorsal dentate gyrus.

Comparison of the effects of TMZ using Ki-67 showed significantly fewer positive number of total cells. A significant decrease was found both in the ventral (*P*<0.05) and dorsal (*P*<0.05) dentate gyrus (Figure 1b). Thus, TMZ treatment resulted in a sustained decrease in cell proliferation in both the dorsal (30.8%, 392 average cells vehicle vs 300 TMZ) and ventral (35.4%, 219 average cells vehicle vs 159 TMZ) dentate gyrus 9 weeks after cessation of TMZ treatment. Quantification of DCX, an immature neuronal marker, revealed a similar effect of TMZ with a significant decrease in the ventral dentate gyrus (*P*<0.05, 2136 average cells vehicle vs 1410 TMZ) that was, however, insignificant in the dorsal dentate gyrus (4241 average cells vehicle vs 3585 TMZ; Figure 1c). Thus, a decrease in neurogenesis, measured using DCX, was also sustained, albeit only in the ventral dentate gyrus (30.6%). In summary, the TMZ treatment used in this study was sufficient to sustain decreased levels of neurogenesis, particularly in the ventral dentate gyrus.

### Acute stress induces a greater increase in corticosterone in mice with TMZ

**Figure 3:**
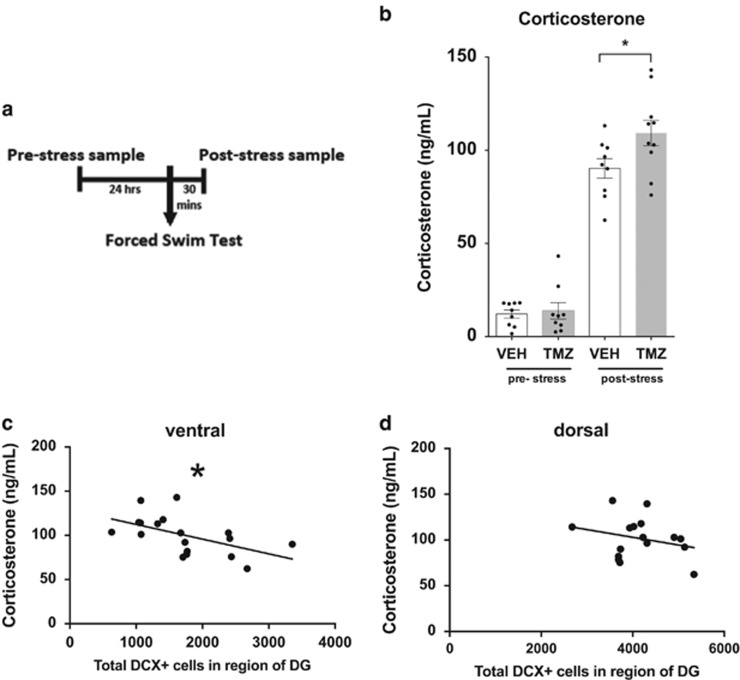
Acute stress induces a greater increase in corticosterone in temozolomide (TMZ)-treated mice. (**a**) TMZ- and vehicle-treated mice were exposed to an acute stress (forced swim) and blood samples were taken both at baseline (pre-stress) and after acute stress (post-stress). (**b**) Baseline levels of corticosterone (pre-stress) showed no significant differences between vehicle- and TMZ-treated mice (*P*>0.05, *n*=9); whereas post stress, TMZ-treated mice show increased levels of corticosterone (*P*<0.05, *n*=9). A significant correlation (*R*^2^=0.2282, *P*=0.0216) was found between the level of neurogenesis and the stress response in the dorsal (**c**) but not ventral (**d**) dentate gyrus (DG) (*R*^2^=0.0701, *P*=0.32). *P⩽0.05.

Stress and depression are integrally linked and, therefore, the potential effects of TMZ-induced decreases in neurogenesis on the biological stress response was examined. Accordingly, the acute corticosterone response was measured in mice exposed to the forced swim test. The blood samples were taken from the same mouse 24 h before the stress exposure and 30 mins after. The results from quantification of corticosterone blood levels in individual mice demonstrated no baseline difference between the different groups (Figure 3a). However, in response to stress, the TMZ-treated mice displayed a significantly greater corticosterone response in comparison with untreated mice (*P*<0.05, Figure 3b). This indicates that TMZ-induced decreases in neurogenesis increases the response to stress. This was further confirmed by examining the relationship between the individual level of ventral neurogenesis (measured using DCX) and the corticosterone levels. The results from this regression showed a significant negative correlation in that the mice with low levels of neurogenesis had a higher corticosterone response (*R*^2^=0.2282, *P*=0.0216, Figure 3c). This was specific to the ventral dentate gyrus as there was no correlation between the dorsal levels of neurogenesis and the corticosterone response (*R*^2^=0.0701, *P*=0.32, Figure 3d).

### TMZ results in a transient reduction in sucrose preference

**Figure 4:**
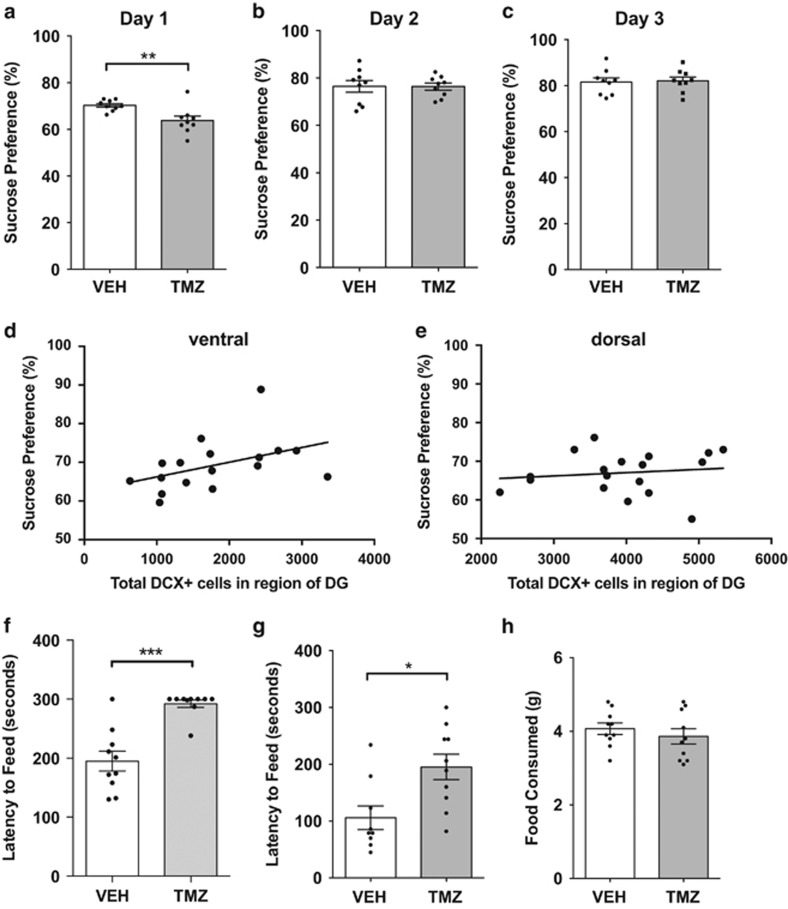
TMZ results in a transient reduction in sucrose preference and results in robust effects in NSF. (**a**) TMZ-treated mice had a significant decrease in sucrose preference on day 1 (*P*<0.01, *n*=10). (**b**) On day 2, there was no difference. (**c**) On day 3, there was no preference as well as no side preference indicating that the results on day 1 were not due to this. (**d** and **e**) Increases of adult neurogenesis in the (**d**) ventral dentate gyrus (DG) are slightly correlated to a higher sucrose preference (day 1; *R*^2^=0.1924, *P*=0.078), but (**e**) there was no correlation present with dorsal DG neurogenesis (*R*^2^=0.0018, *P*=0.592). (**f** and **g**) Mice which have been treated with TMZ have a significant increase in their latency to feed when compared with vehicle-treated mice in two separate cohorts at two different time points. Time points included both directly after TMZ treatment (*P*<0.001, *n*=10; **f**) and after a 6-week recovery period (*P*<0.05, *n*=10; **g**). Food motivation was not affected as there was no difference in food consumed (**h**). *P⩽0.05, ***P⩽0.001. NSF, novelty-suppressed feeding; TMZ, temozolomide.

Anhedonia, a lack of pleasure seeking, is a core, diagnostic symptom of depression and readily modelled using the sucrose preference test. To assess the potential effects of TMZ-induced decreases in adult neurogenesis on anhedonia, the mice were presented with drinking bottles with a 1% sucrose/water solution and plain water, respectively, for a period of 24 h. This was done initially over a period of 2 days where the position of the bottles was switched daily to avoid side preference confounds. The results from the first day of testing demonstrated a highly significant decrease in sucrose preference in the TMZ-treated mice (*P*<0.01, Figure 4a). However, results from the second day show there was no significant difference in sucrose preference between groups (Figure 4b). As this result could be an indication of side preference, a third day of testing was performed. However, results from the third day showed similar results to day 2 indicating no side preference and no anhedonia. These results indicate a transient decrease in sucrose preference when first exposed to the sucrose solution. Individual levels of sucrose preference on day 1 were analysed in correlation with the respective levels of ventral neurogenesis (measured using DCX). The results from this analysis showed that, although not significant, the transient decrease in sucrose preference (day 1) was slightly correlated to a decrease in neurogenesis (*R*^2^=0.1924, *P*=0.078, Figure 4d). The mice with less neurogenesis thus displayed a tendency toward less sucrose preference. This was not the case with dorsal neurogenesis where there was no correlation (*R*^2^=0.0018, *P*=0.592, Figure 4e). The fact that the sucrose preference is only transient indicates that the TMZ-induced decrease in adult neurogenesis alone does not robustly induce anhedonia.

### TMZ results in robust effects in NSF

Examination of NSF—the phenomenon in which exposure to a novel environment suppresses feeding behaviour—in conflict with the increased drive to feeding following food restriction has several aspects which make it relevant to the study of depression. To examine the effect TMZ-induced reduction in neurogenesis may have, two different cohorts of mice were exposed to the NSF test at two different time points. The first time point was immediately after cessation of the TMZ treatment (time point 1, Figure 4f) and the second was after a 6-week recovery period (time point 2, Figure 4g). The results from the time point 1 revealed that TMZ treatment resulted in a highly significant latency to feed in comparison with untreated mice (*P*<0.001, Figure 4f). At this time point, the majority of TMZ-treated mice did in fact not make an attempt to feed within the 5 min test period. No difference was found in food consumption for a 24 h period after the NSF test, indicating no changes in appetite (Figure 4h). However, other factors associated with the TMZ treatment may have affected the NSF at this time point and therefore the effects on hyponeophagia after the recovery period were also examined. Although there was a larger degree of variation in comparison with time point 1, results from time point 2 also revealed a significant increase in the latency to feed in the TMZ-treated mice (*P*<0.05, Figure 4g). Both untreated and treated mice displayed similar weights (data not shown) indicating again that the TMZ treatment did not affect appetite. These results thus show that TMZ treatment results in deficits in hyponeophagia, which are sustained for a substantial time period.

### Anxiety-like behaviour is not robustly affected by TMZ

**Figure 5:**
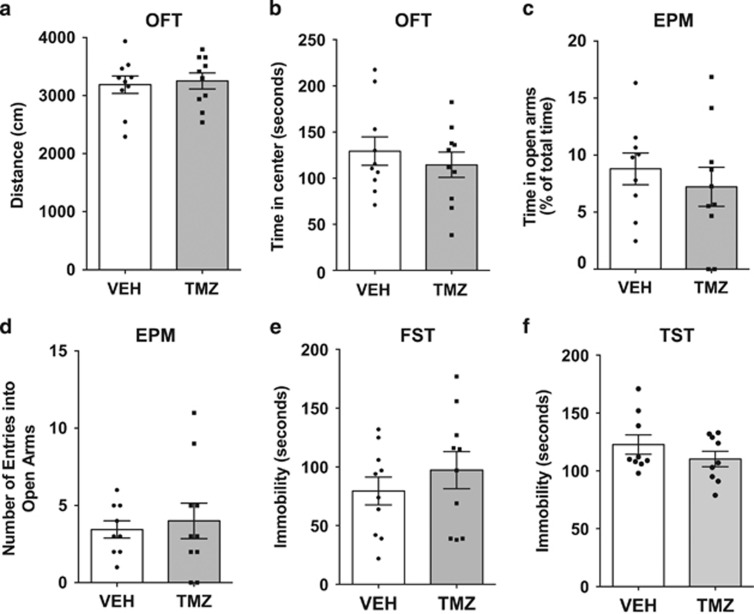
Anxiety-like behaviour and behavioural despair are not robustly affected by TMZ. (**a** and **b**) Open-field test—TMZ treatment does not affect locomotion (**a**) or the time spent in the centre suggesting no effect on anxiety-like behaviour (**b**). (**c** and **d**) Elevated plus maze—this same tendency was reflected in the elevated plus maze where there were no differences in the time spent in (**c**), nor entries into (**d**) the open arms. (**e**) Tail suspension test—no significant differences between treatment groups suggesting no effects on behavioural despair. (**f**) Forced swim test—no significant difference in TMZ mice verifying lack of effect on behavioural despair. EPM, elevated plus maze; OFT, open-field test; TMZ, temozolomide.

A decrease in the latency to feed in the NSF test is often interpreted as an increase in anxiety. To further examine anxiety, analysis of the behaviour of TMZ-treated mice in other paradigms, which more specifically look at anxiety but do not have confounds such as motivation present in the NSF, was performed. These paradigms included the open-field test and elevated plus maze. The analysis of the distance moved showed that there were no significant differences between TMZ-treated and untreated mice, indicating that the treatment did not affect locomotion (Figure 5a). Furthermore, analysis of the time spent in the centre, a measure of anxiety, did not differ between groups indicating that TMZ did not affect anxiety in the open-field test (Figure 5b). This lack of effect on anxiety was confirmed using the elevated plus maze. The results from analysis of behaviour in this test showed no significant differences in the time spent in the open arms nor in the number of entries into the open arms (Figures 4c and d). Therefore, results from the anxiety paradigms demonstrate that TMZ alone does not result in an anxious phenotype.

### Behavioural despair is not robustly affected by TMZ

Behavioural despair or the inability to attempt escape has been suggested to indicate depressive-like behaviour. To investigate the possibility that TMZ-induced decreases in adult neurogenesis may cause an increase in behavioural despair, the mice from both treatment groups were exposed to two behavioural despair paradigms including the forced swim test and tail suspension test. The analysis of data from the forced swim test show that mice treated with TMZ had slightly increased level of immobility (18%) that was though nonsignificant (Figure 5e). This indicates that behavioural despair is not robustly induced by TMZ treatment. Behavioural results from the tail suspension test confirmed this and show in fact a slight decrease in immobility (10%) that was again nonsignificant (Figure 5f). Regression analysis of all behavioural data showed no correlations with levels of neurogenesis, thus further confirming that TMZ-induced decreases in neurogenesis do not appear to be sufficient to induce a depressive-like phenotype.

## Discussion

The results from the different behavioural paradigms show that in mice, TMZ affects several different parameters, which may be relevant to clinical depression. The most robust of these behavioural findings is the effect of neurogenesis depletion on hyponeophagia in the NSF test seen at different time points. Classically the NSF test is often interpreted to be an indicator of anxiety-like behaviour as well as anhedonia though this study as well as previous studies report that despite clear results in the NSF, neurogenesis depletion does not induce anxiety-like behaviour.^26^ In fact, a meta-analysis of studies examining anxiety-like effects related to adult neurogenesis show that, in agreement with the current study, decreases in neurogenesis have no effect on anxiety.^27^ This indicates a deeper complexity of the NSF beyond anxiety and indicates it likely is a measure of other behavioural mechanisms. Indeed, the NSF test is one of the only tests that responds to chronic antidepressant treatment and not acute suggesting that this test engages biological processes, which have parallels to depression and its treatment. Although the nature of these biological processes remains to be confirmed, several recent findings suggest that a central function of adult neurogenesis and even the mechanism central to the NSF test may be in the processing of novel information.^26, 28^ Whether memory processing is important to the development of depression is currently debatable as exemplified in a recent paper in which changes in hyponeophagia were attributed to memory processing, rather than being related to mood, making thus a clear distinction between memory and mood.^26^ In contrast, recently it has been proposed that neurogenesis may have a role in stress-related disorders through its function in contextual emotional processing of memories and that deficits in neurogenesis may contribute to depression via negative assessments of novel contexts thus altering perception.^29^ Accordingly, the memory processing during the NSF may therefore be directly related to mood, rather than being distinct. Therefore, another interpretation is that the robust effects seen in the NSF test indicate that reductions in neurogenesis possibly affect contextual emotional memory processing.

Hyponeophagia is an assessment tool developed for animal models but as described, the mechanistic tangents may be relevant to human depression. Patients undergoing various forms of chemotherapy, similar to TMZ, often present with depressive symptoms suggested to be partly related to decreases in adult neurogenesis.^3^ The emotional cost of cancer itself undoubtedly has a role in the development of depression in patients. Adult neurogenesis cannot be readily measured in living human individuals but the fact that TMZ has such robust effects on neurogenesis using a dosage and treatment cycle in mice that is comparable to the treatment used clinically suggests that patients treated with TMZ may also have depleted levels of adult neurogenesis. It is therefore possible that chemotherapy-induced reductions in neurogenesis also contribute to depression via neurogenesis-dependent processes such as deficits in contextual emotional processing of memories. The implications of this are that patients being treated with chemotherapy may have behavioural deficits that have parallels to those seen in this study.

Another finding relating TMZ-induced changes to depression are the effects on stress. In this study, mice that have been exposed to an acute forced swim stress display an expected increase in corticosterone. However, mice that have depleted neurogenesis as a result of TMZ have an even greater corticosterone response to stress, which is inversely correlated to the individual levels of neurogenesis. A previous high-profile study by Snyder *et al.*^30^ has shown similar findings though this was not correlated to individual levels of neurogenesis. One possible reason this correlation has not been seen before may be the method by which ventral vs dorsal has been sectioned for quantification, which may have led to dorsal regions being quantified as ventral.^31^ The new method of sectioning described here may allow the proper resolution necessary to observe this correlation. Another factor that may affect both levels of neurogenesis and stress levels are the home-cage environment and light phases during which testing was done. Per local regulations, home cages of all the groups in this study were given shelter and nesting materials, which may be interpreted as slightly enriched environment in comparison with other studies not providing shelter or nesting material. Enrichment is known to increase adult hippocampal neurogenesis^32^ and could not only potentially mask even more prominent behavioural and neurogenesis changes in the TMZ groups, but may also similarly alter stress responsivity in control groups. Alternatively, it could be argued that the slightly enriched housing is more representative of a natural environment and may result in more interpretable findings. Regarding lighting, both this study and Snyder *et al.*^30^ were done during the light phase, whereas experiments done during the dark phase have found the opposite effects that neurogenesis impairs the stress response.^33^ Snyder *et al.*^30^ attribute the increase in stress-induced corticosterone response seen in neurogenesis-depleted animals to an impairment of negative feedback on the hypothalamic–pituitary–adrenal axis. Although this mechanism may be a contributing factor to this observed phenomenon, several challenges in direct response to this idea were raised including a lack of GR receptors in young neurons,^34^ and the potential for circuitry-related mechanisms, which theoretically may also cause changes in stress regulation proposed by Gould and colleagues.^35^ Along similar lines of this suggested alternative mechanism,^35^ the changes in stress perception in the theory described above regarding impaired contextual emotional processing may also affect stress reactivity.^29^ For example, human measurements of cortisol levels in response to stress show that individuals with deficits in emotional regulation have a higher cortisol reactivity.^36^ Although there are obvious limitations in rating individual experiences of stress or emotional regulation in animals, corticosterone may similarly be related to emotional regulation and stress reactivity. Therefore, neurogenesis-deficient mice may have deficits in contextual emotional processing causing the observed increase in cortisol to simply be the result of an increased perception of stress. The TMZ-induced changes in corticosterone response again may have important implications for patients treated with chemotherapy as glucocorticoid levels in humans may be similarly affected. Indeed, previous clinical studies indicate dysregulation of the glucocorticoid system in cancer patients treated with different chemotherapy treatments.^37, 38, 39^ Although hormonal stress response reactivity has not been measured in these studies, one study reported changes in diurnal glucocorticoid rhythm in treated patients that was furthermore associated with depression.^39^ Measurements of the acute stress response in this study present only a limited aspect of stress-response regulation as a result of neurogenesis depletion. Clearly these results as well as reported clinical deficits in patients indicate that further investigation is warranted and may be of clinical importance.

The results from the sucrose preference indicate that in this study, anhedonia is affected but only transiently. Previous studies examining the effects of depleted neurogenesis on anhedonia show again contrasting results with neurogenesis depletion resulting in both an increase in anhedonia and no effect.^30, 33^ In the present study, a lack of habituation period, which is however present in the referenced studies, allowed the observance of the transient decrease, which would otherwise have not been observed. This transient period is interesting, nonetheless, as it indicates a neurogenesis depletion-induced difference in behaviour is related to anhedonia. The connection between the observed behaviour and neurogenesis is further supported by the trend seen in the correlation between transient decreased sucrose preference and ventral neurogenesis levels in this study. A similar correlation in whole hippocampus has previously been reported in rats.^40^ Alternatively, however, the reason for this effect may be similar to the observation in the NSF test and is the response of neurogenesis-depleted mice toward novelty, in this case, the presentation of a new taste. Therefore, the transient decrease in sucrose preference may possibly be a result of transient anhedonia, a deficit in novelty processing or a combination of both.

TMZ as an experimental depletion method is relatively inexpensive and is easily injected, making this pharmacological approach a much more available alternative to decrease adult neurogenesis. Some genetic models of neurogenesis ablations are absolute and permanent, whereas TMZ induces an 80–90% reduction which then slowly recovers. An advantage of this is that there is a general uniformity in the level of depletion across groups, whereas other methods which require precise injections will result in highly variable levels of depletion. In our study, the recovery time allowed a substantial recovery of 6 weeks yet robust behavioural effects were still present while neurogenesis deficits were still present after 9 weeks of recovery. This indicates that total ablation is not necessary to observe biological and behavioural changes and that in fact, such depletion levels are likely more translational and more representative to the clinical scenario. In addition, methods inducing a total ablation may initiate compensation mechanisms, which may lead to misinterpretation of results. However, a specific disadvantage of TMZ depletion is the global nature of the depletion. Some depletion methods allow a spatial specificity targeting only the hippocampus, whereas TMZ also affects the other neurogenic niche as well as other types of cell division. Although TMZ has less side effects than other antimitotic compounds, it still has the potential to affect other tissues. This was in a previous study, however, found to be minimal and a non-issue after sufficient recovery time.^7^ Previous studies have also demonstrated that TMZ affects certain aspects of cognition, particularly spatial and associative learning.^7, 11^ Although these types of cognition are unlikely to affect the parameters measured in this study, it cannot be excluded that other types of potential cognitive deficits due to a decrease in adult neurogenesis may have an effect on a depressive-like phenotype. Indeed, cognitive deficits in themselves are considered to be an important aspect of depression.^41^

As TMZ is the standard treatment for cancers of the brain, thousands of patients are treated with this drug. In addition to its utility as an experimental tool in animal models, studying the effects of TMZ has important translational implications for all patients being treated with chemotherapy. Although it is not possible to experimentally deplete adult neurogenesis in humans, this study gives strong evidence that chemotherapy-induced decreases in adult neurogenesis may affect the stress response and behaviours related to processing novelty, both of which ultimately may contribute to depressive symptoms. In addition to TMZ, the antimitotic action of other cancer drugs has already been demonstrated in animal models to decrease neurogenesis and affect spatial memory,^42^ though these do not affect neurogenesis-independent functions such as fear conditioning and novel object recognition.^43^ Although no changes in depressive-like behaviour have been noted in other studies, it is likely that other types of chemotherapy may similarly affect behaviours and biological parameters, which have the potential to contribute to depression. Despite their potential side effects, TMZ and other forms of chemotherapy are indispensable for increasing patient-survival rates. However, future studies, which harness the benefit of doubt of these antimitotic drugs and examine their effects on neurogenesis depletion may yield new insights into neurogenesis function in humans, an area of study that is otherwise highly in need for translational verification. Studying the specific effects of chemotherapy on mood may also initiate important changes in prophylactic and post-chemotherapy treatments, which may increase the quality of life for those affected.
